# Autopsy-based retrospective study of hanging deaths at hospital center in Central India

**DOI:** 10.6026/973206300222296

**Published:** 2026-04-30

**Authors:** Vishal Sean Baweja, Vivek Kumar Chouksey, Vaibhav Agrawal, Narendra Singh Patel

**Affiliations:** 1 Department of Forensic Medicine and Toxicology, People's College of Medical Sciences & Research Centre, Bhopal, Madhya Pradesh, India; 2 Department of Forensic Medicine and Toxicology, ABVGMC, Vidisha, Madhya Pradesh, India

**Keywords:** Hanging, suicide, autopsy, forensic medicine, epidemiology, ligature material

## Abstract

Hanging is one of the most common methods of suicide in India, particularly among young adults. Hence, this retrospective autopsy-based study was conducted on 39 cases of death by hanging at Atal Bihari Vajpayee Government Medical College, Vidisha, over a two-year period. The majorities of victims were males (62%) and aged 21-30 years (43.58%). A significant proportion were married (59%) and most incidents occurred indoors (87.17%), indicating a preference for privacy. This study advances knowledge by providing regional data on hanging-related suicides, highlighting the role of social, psychological and familial stressors and emphasizing the need for early psychological intervention and proper handling of crime scenes in suicide prevention.

## Background:

An autopsy is a necessary part of the evidence-gathering procedure for any legal investigation into unnatural (homicide, suicide, accident), suspicious or unexpected deaths [1]. Determining whether a death was accidental, suicidal, homicidal, or natural is crucial because asphyxial death is a frequent occurrence in forensic practice. While the scene investigation and evidence collection have their own relevance, a through and precise autopsy is crucial to solving the case in such deaths [2]. Suffocation (environmental, smothering, choking, mechanical and suffocating gasses), hanging, strangulation (manual and ligature), chemical asphyxia (carbon monoxide (CO), hydrogen cyanide and hydrogen sulfide) and drowning are some of the ways that asphyxial fatalities can occur [3]. Hanging is responsible for a considerable percentage of suicidal deaths (57.8%). Suicide by hanging is the most prevalent method in poor nations such as India [4]. The gravitational pull of some or all of the body's weight causes constriction of the neck structures in hanging, a type of ligature strangling [5]. There are two varieties of hanging that are recognized: complete hanging, which involves the victim's entire body suspended freely and incomplete hanging, which involves a portion of the body bearing the victim's weight [6]. Therefore, it is of interest to describe the demographic patterns and medico-legal characteristics associated with deaths due to hanging.

## Materials and Methods:

This is a retrospective study that included all cases with an alleged history of hanging brought to the mortuary in Department of Forensic Medicine and Toxicology at Atal Bihari Vajpayee Government Medical College, Vidisha, for autopsy between January 2022 and December 2023.

## Inclusion criteria:

[1] All confirmed cases of death due to hanging subjected to autopsy during the study period

[2] Cases with complete autopsy records and relevant police inquest reports

## Exclusion criteria:

[1] Decomposed bodies where definitive opinion regarding hanging could not be established

[2] Cases with incomplete records

[3] Cases of strangulation or other forms of asphyxia

Meticulous autopsy examinations were performed in all cases and findings were carefully documented in a structured format. The details pertaining to the epidemiological profile were obtained by means of the information provided by the relatives and/or the hospital records.

## Statistical analysis:

Data were entered in Microsoft Excel and analyzed using appropriate statistical software SPSS version 25. Descriptive statistics such as frequency, percentage, mean and standard deviation were calculated. Chi-square test was applied to assess associations between variables. A p-value <0.05 was considered statistically significant.

## Ethical considerations:

Permission was obtained from the Institutional Ethics Committee prior to conducting the study. Confidentiality of personal information was strictly maintained. As the study was retrospective and based on autopsy records, no direct consent was required.

## Results:

A total of 494 post mortems were done between January 2022 and December 2023. Out of these, the cases of hanging comprised of 12 in 2022 and 27 in 2023 making it a total of 39 (7.89%) cases. The maximum number of hanging cases were seen in the month of July (17.94%), followed by September and October respectively (12.82%), as in depicted in Table 1 (see PDF). The male victims outnumbered the female victims in both the years with 10 (83.33%) in 2022 and 14 (51.85%) in 2023; the male to female ratio coming out as 5:1 and 1.07:1 in 2022 and 2023 respectively. Overall, male victims comprised of 62% deaths as opposed to 38% females, as has been depicted in Figure 1 (see PDF). The majority of victims belonged to the 21 30 years (43.58%) age group in both 2022 and 2023 followed by the fourth decade (23.07%), as in depicted in Table 2 (see PDF). The youngest deceased was of age 7 years and the eldest of age 56 years. Among all cases in the study period, 23 (59%) were married and the remaining were unmarried, as is depicted in Figure 2 (see PDF). The place of incidence was predominantly indoors, 34 (87.17%) and outdoors in the remaining cases, with 2 cases wherein the place of incidence was a toilet that was constructed away from the residence but did not have a proper door. The remaining outdoor cases of hanging were either on self-owned farming lands or where the deceased worked as a farmer, depicted in Figure 3 (see PDF). Body was taken down by the relatives in majority of the cases, 27 (69%), whereas in the remaining cases it was taken down in the presence of police personnel, as depicted in Figure 4 (see PDF). History of previous suicidal attempts and/or evidence of previous suicidal attempt were found to be present in 9 (23%) cases. The ligature material was found in-situ in 18 (46.15%).

## Discussion:

In our study, the age group 21-30 years was found to be the most prevalent. This can be attributed to the fact that this age group has to bear the maximum brunt of the modern social disorganization, increased competition in all fronts of life largely pertaining to the difficult professional survival. This paired with the usual responsibilities like those of family, ageing parents and liabilities adds to a lot of stress. Several authors have reported the 21-30 years age group as the most commonly involved age group [7, 8, 9-10]. This finding is inconsistent with the findings of Elfawel and Awad [12] found the most common age group to be 30-39 years. In our study the male sex was found to be more commonly involved with 83.33% cases in 2022 and 51.85% cases in 2023 as males. Overall, male victims comprised of 62% deaths as opposed to 38% females across the study duration. This finding can be attributed to the societal roles that males are more commonly known to fulfill, like earning the livelihood, looking after the family, professional competition etc. In this way the male sex usually bears more mental stress and agony which could be the reason why male sex has found to be more commonly resorting to hanging. This finding concurs with the findings noted by other authors like Mishra *et al.* [11] and Das *et al.* [13] in their studies. The marital status is also a critical epidemiological factor; in our study most of the cases were found to be married, similar findings were observed in the study conducted by Ali *et al.* [14], Gouda and Rao [15] reported that the majority of the cases were married. The higher number of cases who were married and died due to hanging can be attributed to the additional burden of looking after family over and above the professional and personal responsibilities of a person. This finding is in contrast with the observed by Chavan *et al.* [16] in which unmarried cases outnumbered the married cases. The place of hanging was predominantly indoors in our study which concurs with the findings of Jain *et al.* [7]. The prevalent choice of indoor location for hanging seems to be suggesting that the victim did not want to take the chance of being noticed which more likely outdoors. The history and/or evidence of previous suicidal attempt is a crucial factor that highlights the tendency of a person and possibly points towards an underlying unresolved or partially resolved psycho-social issue. In our study 23% cases had such evidence while in a study conducted by Chavan *et al.* [16], it was reported that approximately 9% of the victims of suicidal deaths had history of attempting suicide in the past. Among all the autopsies conducted in the current study, ligature material was brought in situ with the body in 46.15% cases. This can be due to the fact that often the relatives bring the body down and do not carry the ligature material with them when they bring the person to the hospital. Küçük *et al.* [17], in an autopsy-based study of childhood hanging deaths, emphasized that the epidemiology of hanging is strongly influenced by the demographic profile of the study group. Usually when the body is taken down in the presence of police personnel, the ligature material is either left in-situ or is carried along with the body. The police personnel understand the importance of ligature material where they have been given proper training and this highlights the importance of proper training to improve the quality of post mortem examination that is being done and for the proper delivery of justice to the aggrieved.

## Conclusion:

Suicide is a serious and growing public health problem. It is caused by many factors, with psychological stress being one of the main reasons. Early mental health support, better awareness, and community help can reduce such deaths.
